# Hybrid microwave–infrared thermal desorption for remediation of oil-contaminated offshore drill cuttings: field-sample evaluation and process performance

**DOI:** 10.1007/s11356-026-38229-2

**Published:** 2026-09-24

**Authors:** Roohollah Babaei-Mahani, Ali Fereidounpour, Yinghe He, Peter Scholes

**Affiliations:** 1 Department of Chemical Engineering, Brunel University London, Uxbridge, UK; 2 Brunel University of London, Uxbridge, England

**Keywords:** Drill cuttings treatment, Microwave/infrared processing, Environmental risk mitigation, Waste management, Drill cuttings processor, Offshore ecosystem protection

## Abstract

Drilling operations generate large volumes of drill cuttings contaminated with hydrocarbons, salts, and trace metals, creating significant environmental and regulatory challenges for offshore disposal. Most regulatory frameworks prohibit the discharge of drill cuttings containing more than 1 wt.% oil-on-cuttings, necessitating effective on-site treatment technologies. This study evaluates a novel continuous hybrid microwave–infrared thermal desorption process, the Advanced Thermal Processor (ATP), which, unlike conventional batch thermal desorption systems, simultaneously applies microwave volumetric heating and infrared surface heating within a rotating drum under continuous feed operation. Six samples of offshore water-based drill cuttings contaminated with sour crude oil were collected from Middle Eastern drilling operations and processed in two batches using a pilot-scale ATP unit under inert, low-oxygen conditions. The treatment was carried out at a solid discharge temperature of 60–100 °C and a residence time of approximately 15–30 min. The process achieved oil removal efficiencies of 95.0–97.8%, consistently reducing oil-on-cuttings to below 1 wt.%. Total Petroleum Hydrocarbons (TPH) and Total Oil and Grease (TOG) were reduced by more than 96% across all samples. Thermogravimetric analysis confirmed that most volatile contaminants were removed at temperatures below 110 °C, suggesting a substantially lower thermal demand than conventional incineration or friction-based thermal desorption, which typically operate above 300–500 °C. The results demonstrate that hybrid microwave/infrared thermal processing is an effective and energy-efficient approach for the treatment of offshore drill cuttings, enabling regulatory compliance while minimising secondary emissions and operational disruption.

## Introduction

### Background and context

Drill cuttings generated during oil and gas exploration represent a significant environmental challenge, with volumes often exceeding 5000 m^3^ per well (Biltayib et al. 2016). These wastes consist of fragmented rock contaminated with drilling fluids and additives, including hydrocarbons and heavy metals, which pose risks to soil and water systems if not properly managed.

The quantity and characteristics of drill cuttings depend on factors such as formation properties, well depth, borehole diameter, and drilling fluid type. In particular, oil-based and synthetic-based fluids increase contamination levels, while borehole washout and weak formations can significantly amplify waste volumes. These factors complicate treatment and disposal, necessitating efficient and environmentally sound management strategies.

Current drill cuttings management methods, including landfarming, subsurface injection, biodegradation, solidification, and incineration, are associated with important limitations. Biological approaches are often slow and ineffective for heavily contaminated materials, while landfarming is restricted to low-toxicity wastes. Subsurface injection transfers contaminants to deeper formations, raising long-term environmental concerns. Solidification does not fully eliminate leaching risks, and incineration, although effective, is energy-intensive and may generate secondary emissions. These limitations highlight the need for more efficient and sustainable treatment technologies.

Thermal desorption has emerged as a promising approach for hydrocarbon removal. Microwave-based heating, in particular, offers rapid and volumetric energy transfer, enabling efficient contaminant removal (Pereira et al. 2018). However, most studies are limited to batch-scale systems and face challenges related to heating uniformity, energy efficiency, and scalability for continuous industrial applications (Mota et al. 2018).

Despite these advances, there remains a lack of treatment systems that simultaneously achieve efficient hydrocarbon removal, uniform heating, and continuous large-scale operation. To address this gap, this study proposes a hybrid microwave-infrared thermal desorption system for the treatment of drill cuttings. The integration of microwave and infrared heating is designed to enhance heat distribution and process efficiency while enabling scalable operation. The objectives of this work are to (i) evaluate the performance of the hybrid system in hydrocarbon removal, (ii) compare its efficiency with conventional thermal treatment methods, and (iii) assess its potential as a more sustainable solution for drill cuttings management. The proposed approach aims to improve waste treatment efficiency and reduce environmental impact in drilling operations.

### Literature review

Microwave technology has emerged as a highly effective method for treating drill cuttings contaminated with synthetic and oil-based drilling fluids. This technology leverages microwaves’ unique ability to heat materials volumetrically, providing rapid and uniform heating that is not achievable with conventional thermal methods.

Pereira et al. (2018) demonstrated that microwave radiation could reduce n-paraffin content in cuttings contaminated with non-aqueous fluids (NAFs) to below the discharge limit of 6.9% by mass, making it a viable option for on-site decontamination.

Similarly, Mota et al. explored the kinetics of batch microwave drying of drill cuttings contaminated with an olefin-based fluid and achieved significant reductions in drying time and energy consumption, particularly at lower power settings (Mota et al. 2018). Their studies have shown that microwave heating promotes selective heating at the molecular level, providing advantages such as energy efficiency and shorter drying times. For instance, microwave drying technology allows the reuse of the recovered organic phase in the preparation of a new load of drilling fluid, as it does not alter the chemical composition of the fluid.

Microwave heating produced more porous solid residues that enhanced heat and mass transfer during the treatment process, leading to greater mass loss and lower residual oil content in the treated cuttings (Celik et al. 2019). Several studies have compared microwave and infrared technologies to conventional thermal methods such as electric heating. Microwave heating has been shown to produce more porous solid residues, which enhance heat and mass transfer during the treatment process. This leads to greater mass loss and lower residual oil content in the treated cuttings (Junior et al. 2019).

In contrast, electric heating relies on the thermal conductivity of the material and the heat transfer efficiency in the system, which can be less effective for decontaminating drill cuttings. Additionally, the use of microwave technology has shown that it can promote the pyrolysis of petroleum hydrocarbons, making it more suitable for treating oil-based drilling cuttings (Hou et al. 2018).

Microwave technology offers a promising solution for the treatment of drill cuttings contaminated with synthetic and oil-based fluids. Its ability to provide rapid, uniform heating with reduced energy consumption and environmental impact makes it a superior alternative to traditional thermal methods. However, challenges such as scaling up the technology for continuous processing need to be addressed to fully realise its potential in industrial applications. Further research into the optimization of process parameters and the design of continuous microwave treatment systems is necessary. The integration of microwave technology with existing oil and gas operations can significantly reduce the environmental footprint of drilling activities and provide a sustainable method for the remediation of drill cuttings.

### Regulatory bodies

Strict environmental regulations control the discharge of drill cuttings, with a primary focus on marine ecosystem protection. The Oslo-Paris (OSPAR) Convention sets the most restrictive standards for offshore activities in the North Sea and North-East Atlantic. Since 2001, OSPAR has prohibited offshore discharge of cuttings contaminated by oil-based and synthetic-based fluids unless residual oil is below 1% by dry weight. In 2012, OSPAR added stringent requirements for sediment monitoring and hydrocarbon reporting at drilling sites to support benthic ecosystem recovery. Other frameworks, including the EPA Effluent Guidelines (USA), Barcelona Convention (Mediterranean), and London Protocol, regulate discharge limits and treatment methods, but OSPAR enforces the strictest, most comprehensive control of offshore discharge practices.

### Drill cuttings volume

Today, oil and gas production as the world’s primary energy source remains a crucial focus for production companies. Accessing oil and gas reservoirs at various depths is associated with producing a large volume of drilling cuttings, which are often contaminated due to the addition of organic and/or inorganic chemicals to the drilling fluid. This also happens while drilling in oil and gas reservoirs. These polluted drilling cuttings can cause significant environmental and public health issues and cannot be discharged into the environment without treatment. The studies show that the returned drilling fluids contain heavy metals like nickel, mercury, cadmium, chromium, copper, and lead. These elements are associated with chronic diseases, including cancer (Antia et al. 2022).

**Fig. 1:**
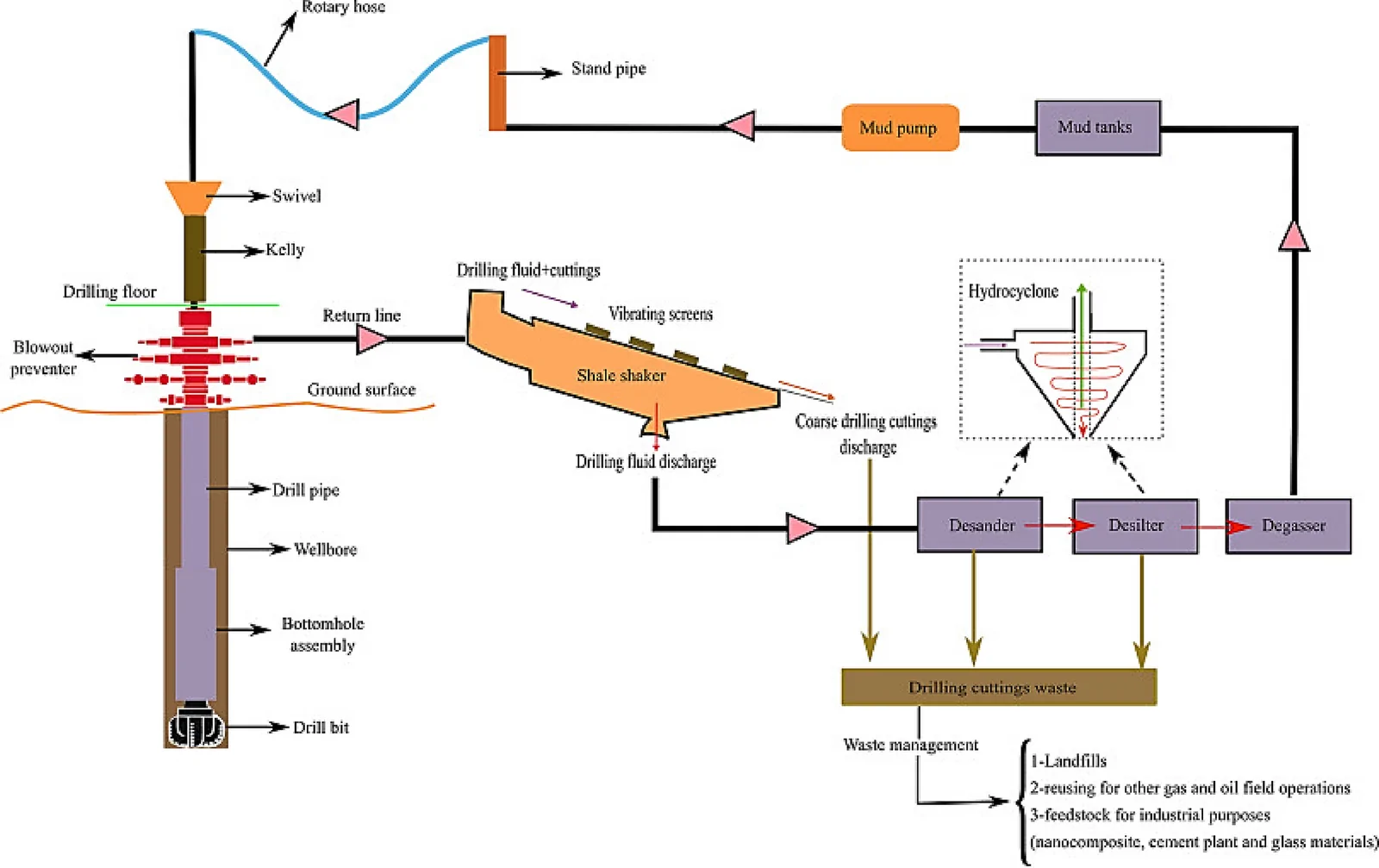
Schematic diagram of a drilling fluid circulation system in rotary drilling operations

The drilling fluid system is one of the main parts of the drilling operation and is the heart of the drilling operation. Figure 1 (Tabatabaei et al. 2022) shows a schematic of the drilling fluid circulation system. Drilling fluid can undertake more than 15 critical functions depending on different situations, from providing the necessary pressure to control formation pressures and overburdens, cooling and lubricating the drill bit and drill string, carrying cuttings to the surface, achieving geological information, etc. Diesel or chemical additives are usually used to impart lubricating properties to the mud. These additives are often derived from oil. The amount of this diesel used is proportional to the need, ranging from 1 to 3 percent of the volume of the drilling mud. On the other hand, adding other chemical additives and polymers makes the drilling cuttings sufficiently contaminated that they cannot be disposed of on land or sea without treatment.

Various methods have been proposed and implemented for treating drill cuttings, each with its advantages and disadvantages. Treatment methods include land spreading, landfarming, bioremediation, reinjection, fixation with agents like cement, thermal, etc. Ilinykh et al. (2023) developed a life cycle assessment (LCA) of drilling waste management methods in Russia. They compared land spraying, disposal, solidification, and reinjection. The results illustrate that solidification has the highest impact due to cement and lime consumption, while reinjection has the lowest environmental impact.

In sensitive environments, especially marine environments, the methods above have not been able to reduce the contamination levels of drilling cuttings below global standards. As a result, specialists have always been looking for new methods to treat contaminated drill cuttings. One of these methods is pyrolysis, which involves the absorption or separation of contaminants from the surface of the drill cuttings by heating them. This method has gained attention in recent years. In the pyrolysis method, researchers have compared the use of conventional heat sources, such as electrical or microwave energy, multiple times. In all these studies, the superiority of microwave techniques over other thermal methods has been proven (Beneroso et al. 2015; Lu et al. 2012; Hossain et al. 2011; Robinson et al. 2008b; Shang et al. 2007). The main reason for this result is the selective and volumetric heating provided by microwave methods.

In all previous and current methods of treating drill cuttings, the treatment capacity has always been a challenge, particularly when drilling the upper sections of the well (top holes), where a large volume of drill cuttings, along with the returning drilling fluid, is discharged onto the shale shaker screens. Equation 1 shows how much cutting is produced during the drilling operation.

1 $$Vc=\frac{\left(1-\phi\right)\ast\left(D+W\right)^2\ast ROP}{1029.4}$$

where

- Vc is the volume of cutting in bbl/hr.
- Ø is formation porosity (%).
- D is the wellbore diameter in inches.
- W is wellbore washout in inches
- ROP is the rate of penetration in feet per hour.

In other words, the production rate of drill cuttings in the upper sections of the well is much higher than their treatment rate. In onshore drilling, due to the availability of sufficient space, these drill cuttings are usually stored in constructed pits like coral, waste pits and tanks, where they are treated over time and eventually disposed of in landfills. Due to the lack of space in offshore drilling platforms, using a sufficient number of skips to store the drill cuttings is impossible. Therefore, there is a need to address this deficiency in the treatment methods and design the methods and related equipment to allow the produced cuttings to be treated quickly on-site and discharged into the sea.

Alternative solutions for handling offshore cuttings usually come with secondary pollution and require significant time and cost. For example, transporting the drill cuttings to shore (skip and ship) for treatment and disposal involves challenges such as high transportation costs, the long distance between the drilling platform and the shore, and air and noise pollution caused by ship traffic. Additionally, disposing of drill cuttings onshore often leads to soil and groundwater contamination.

## Materials and methods

### Process description and experimental system

**Fig. 2:**
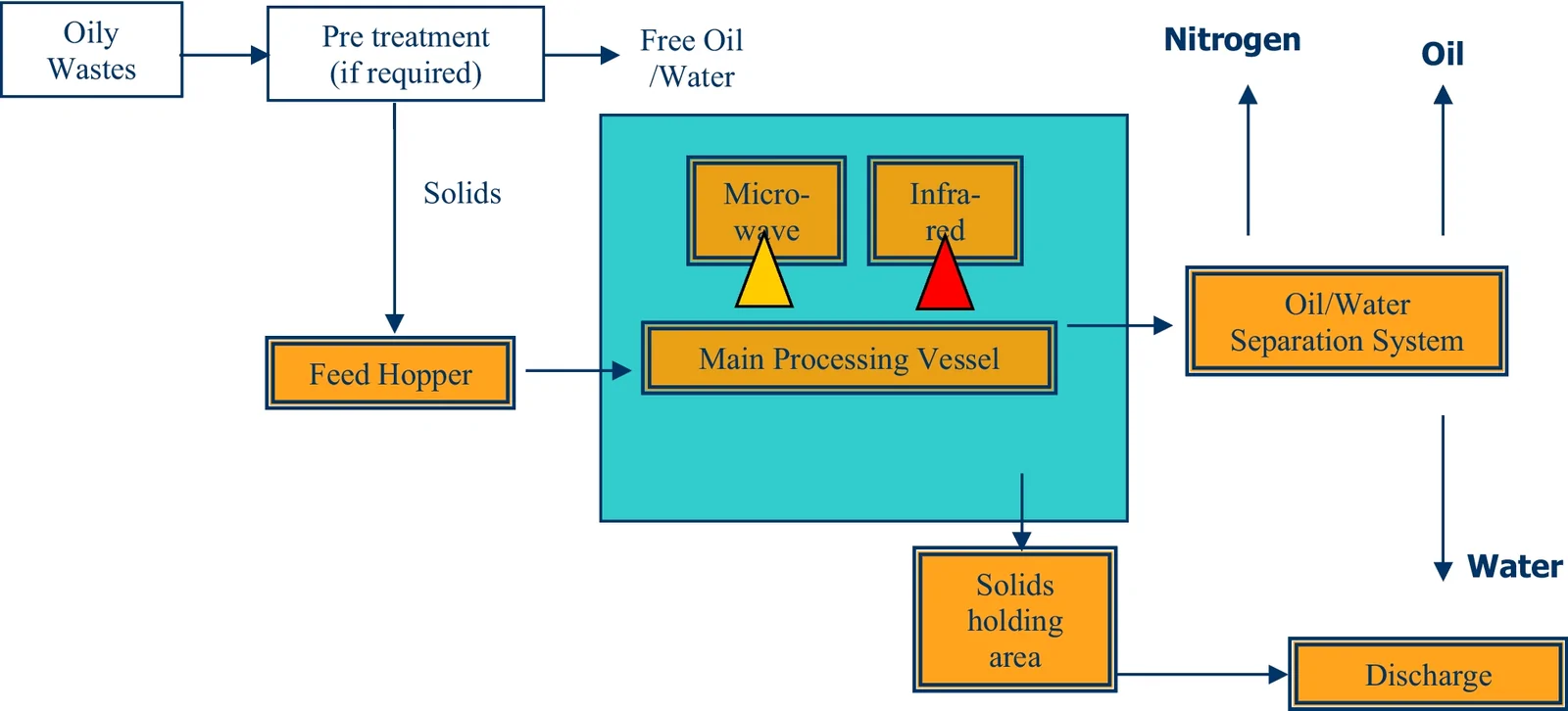
Process flow diagram of the oil and water vapour handling and separation system for contaminated drill cuttings

A continuous hybrid microwave-infrared thermal desorption (ATP) system was employed to treat oil-contaminated drill cuttings. The system comprises a feed unit, a rotating thermal processor, a vapour handling and condensation system, and a discharge unit (Fig. 2). The overall process was designed to operate under oxygen-limited conditions to ensure safe and efficient thermal treatment.

Contaminated drill cuttings were continuously fed into the system via a sealed hopper and conveyed to the processor by a screw conveyor. The feed system was designed to maintain a consistent material flow while minimising air ingress. To accommodate variations in feed properties, including moisture content and flow behaviour, the system incorporated mechanical agitation and controlled feeding mechanisms to ensure uniform delivery of material into the processing unit.

### Feed preparation and pre-treatment

Prior to thermal treatment, the drill cuttings were pre-conditioned to improve process efficiency. A decanting centrifuge was used to remove free water, thereby reducing moisture content and increasing the system’s effective throughput. The dewatered material was subsequently homogenised in a blending unit to ensure a consistent feed composition. The prepared feed was then conveyed into the processor hopper, where a controlled feeding mechanism ensured continuous and stable material input. The hopper design also served as an airlock, helping maintain an inert processing environment.

### Thermal desorption process

Thermal treatment was carried out in a slightly inclined rotating drum, which facilitated continuous material transport and mixing. The residence time of the drill cuttings within the processor was approximately 15 min. During this period, the material was subjected to simultaneous microwave and infrared heating.

Microwave energy provided volumetric heating of the material, while infrared heaters, positioned along the length of the drum, ensured uniform surface heating. This combined heating approach enhanced heat distribution and promoted efficient desorption of hydrocarbons and moisture. The process was conducted at temperatures below 110 °C under inert conditions, maintained through nitrogen purging and controlled pressure differentials within the system.

### Vapour handling and condensate recovery

Volatilised hydrocarbons and water vapour generated during the thermal process were continuously extracted from the processor and directed to a vapour handling system. Initially, the vapour stream passed through a gas scrubber to remove entrained solid particles. It was then cooled in a condensation unit, where oil and water were separated from the gas phase.

The condensed liquid was transferred to an oil–water separator, allowing recovery of oil suitable for reuse and water with residual oil content in the range of 0–15 ppm. Non-condensable gases were recirculated back into the system as a carrier gas, thereby improving process efficiency and reducing emissions.

### Discharge and post-treatment handling

**Fig. 3:**
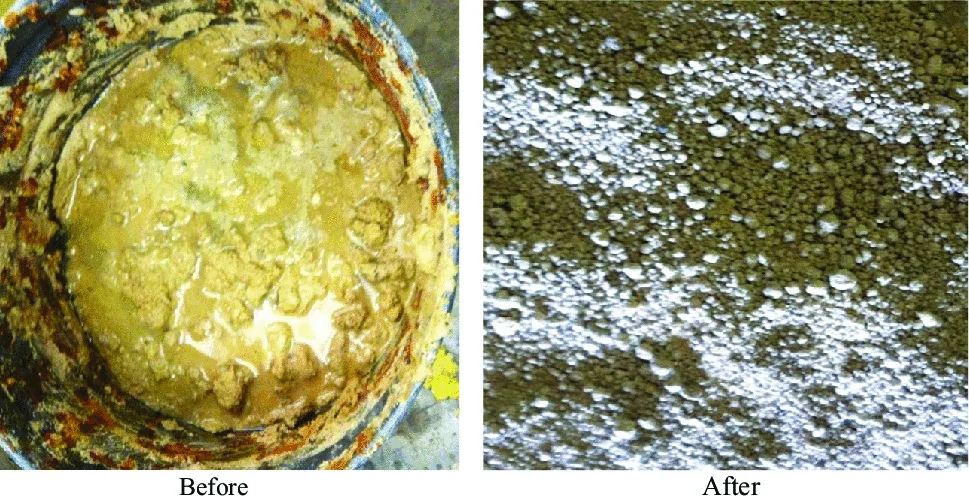
Visual comparison of drill cuttings before and after treatment in the discharge system

Following thermal treatment, the drill cuttings were discharged from the processor through a sealed screw conveyor and rotary airlock valve. The treated material was dry and powder-like, enabling easy handling and transport. A visual comparison of the drill cuttings before and after treatment is shown in Fig. 3, highlighting the significant change in texture and appearance. To minimise dust emissions during discharge, a water mist system was applied. The treated solids were considered suitable for offshore disposal in compliance with regulatory requirements, based on the achieved reduction in oil content.

### Safety and process control

The system was designed to operate under an inert nitrogen atmosphere to prevent combustion and ensure safe operation in an ATEX Zone 2 environment. Oxygen levels were continuously monitored, and automatic safety protocols were implemented to shut down the system in the event of abnormal conditions. A slight positive nitrogen pressure was maintained within the system to prevent air ingress. Additional safety features included controlled gas extraction and emergency nitrogen purging.

### Prototype development and experimental procedure

**Fig. 4:**
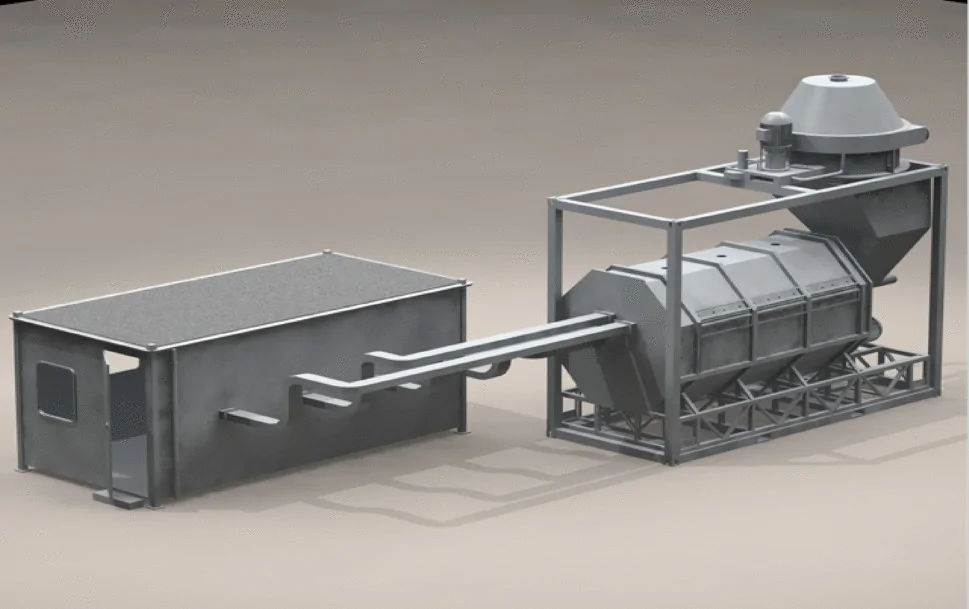
Prototype ATP unit used for vapour handling and condensate recovery in this study

The experimental work was conducted using a pilot-scale prototype developed following initial laboratory-scale testing. The prototype system (Fig. 4) incorporated all major process components, including feeding, thermal treatment, vapour handling, and discharge units. All experiments were performed under controlled operating conditions, and the resulting treated solids and condensates were collected for subsequent analysis.

## Results and discussion

### Sample description and experimental context

Water-based drill cuttings contaminated with sour crude oil were collected from an offshore drilling operation in the Middle East. Six representative samples were characterised and subsequently treated using the pilot-scale hybrid microwave-infrared ATP prototype under controlled laboratory conditions. The objective of this study was to evaluate the performance of the hybrid thermal desorption system and define key operating parameters relevant to full-scale deployment. All samples were processed under standard water-based mud conditions, with operating parameters adjusted to optimise energy absorption and ensure effective contaminant removal.

### Treatment performance and contaminant removal

The hybrid microwave-infrared process achieved consistently high contaminant removal across all samples (Table 1). Oil removal efficiencies ranged from **95.0% to 97.8%**, reducing oil-on-cuttings to below the regulatory threshold of **1 wt.%** in all cases. Similarly, Total Petroleum Hydrocarbons (TPH) and Total Oil and Grease (TOG) were reduced by more than **96%**, demonstrating effective removal of hydrocarbon contaminants.

**Table 1:** Oil, water, and hydrocarbon content of water-based drill cuttings before and after treatment using the ATP prototype unit

Sample ID	Oil content (% W/W)		Water content (% W/W)		Oil removal efficiency (%)	TPH (mg/kg)		TOG (%)	
	Before	After	Before	After		Before	After	Before	After
1	6.70	0.16	17.20	0.00	97.37	15,067.00	590.00	4.62	0.15
2	6.30	0.16	17.50	0.18	97.17	14,119.00	864.00	4.18	0.14
3	6.20	0.17	17.50	0.06	98.73	12,783.00	490.00	3.67	0.11
4	6.90	0.14	16.50	0.10	97.53	17,340.00	661.00	5.80	0.22
5	6.30	0.14	16.30	0.20	97.76	19,740.00	731.00	6.00	0.25
6	4.40	0.22	24.00	0.36	95.00	9500.00	205.00	3.67	0.04

*TPH*, Total Petroleum Hydrocarbons; *TOG*, Total Oil and Grease

Total Petroleum Hydrocarbons (TPH) and Total Oil and Grease (TOG) are critical parameters used to evaluate hydrocarbon contamination in drill cuttings. TPH quantifies the total concentration of petroleum-derived hydrocarbons, while TOG encompasses both petroleum hydrocarbons and other solvent-extractable organic compounds such as animal or vegetable oils. The oil content, water content, Total Petroleum Hydrocarbons (TPH), and Total Oil and Grease (TOG) values reported in Table 1 were obtained from the analytical records for the WBM experimental programme. As the experimental programme was conducted a few years prior to the present study, the available records did not provide sufficient information to verify the individual analytical protocol numbers used for these determinations. Reducing TPH and TOG levels is a key objective, as it directly influences environmental safety, regulatory compliance, and the viability of waste management strategies for treated cuttings. High removal efficiencies achieved through the microwave-infrared treatment process demonstrate its effectiveness in producing near-inert solid waste, thereby supporting safe discharge or potential reuse of the treated material.

Water removal was also highly efficient, with most samples showing near-complete moisture reduction. Slightly lower removal efficiency was observed for Sample 6, which had the highest initial water content (24% w/w), indicating that initial moisture content influences overall process performance. These results confirm that the hybrid thermal desorption process can produce treated solids that meet environmental discharge standards while simultaneously recovering hydrocarbons.

**Fig. 5:**
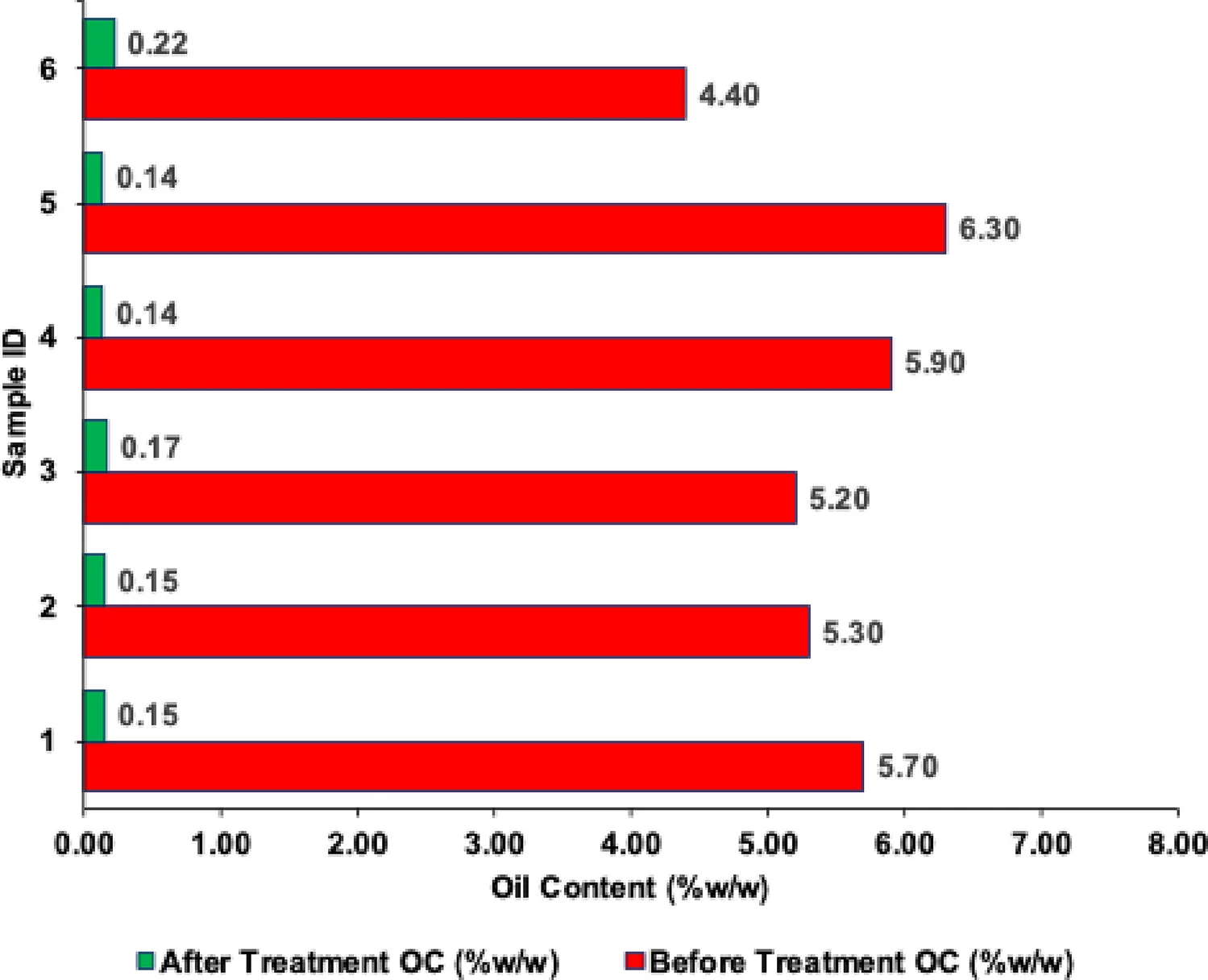
Comparison of oil content in water-based drill cuttings before and after treatment using the ATP prototype unit

Figure 5 presents the reduction in oil content before and after treatment. The results demonstrate that the ATP system effectively removes multiple contaminants, including oil, water, and hydrocarbons, in a single processing step. This integrated removal capability represents a significant advantage over conventional treatment methods, which often require multiple stages. The ability to simultaneously treat different contaminant phases improves both operational efficiency and environmental performance, making the process well-suited for offshore applications.

### Effect of initial water content on oil removal efficiency

**Fig. 6:**
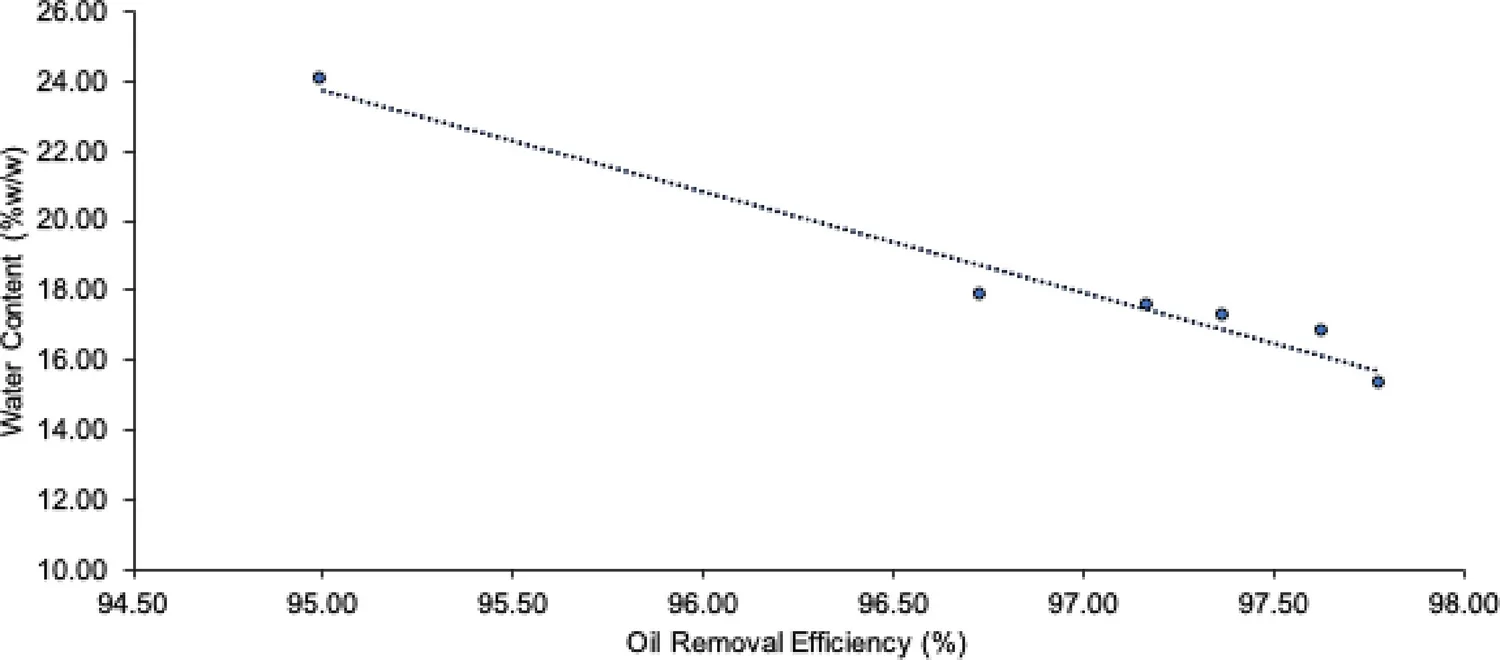
Relationship between initial water content and oil removal efficiency for water-based drill cuttings treated using the ATP prototype

The relationship between initial water content and oil removal efficiency is presented in Fig. 6. A clear inverse trend is observed, with higher water content leading to slightly reduced oil removal efficiency. For example, Sample 6, with the highest water content (24% w/w), exhibited the lowest removal efficiency (~ 95%), whereas samples with lower moisture content achieved efficiencies approaching 97.8%.

This behaviour can be explained by water’s preferential absorption of microwave energy. As a highly polar molecule, water absorbs microwave radiation more efficiently than hydrocarbons, thereby increasing the energy required for evaporation. Consequently, less energy is available for oil desorption in samples with higher moisture content.

In addition, water may form a transient barrier around oil droplets, limiting heat transfer and delaying volatilisation. These findings highlight the importance of feed pre-conditioning, particularly moisture reduction, to optimise treatment efficiency.

### Influence of total fluid content (oil + water)

**Fig. 7:**
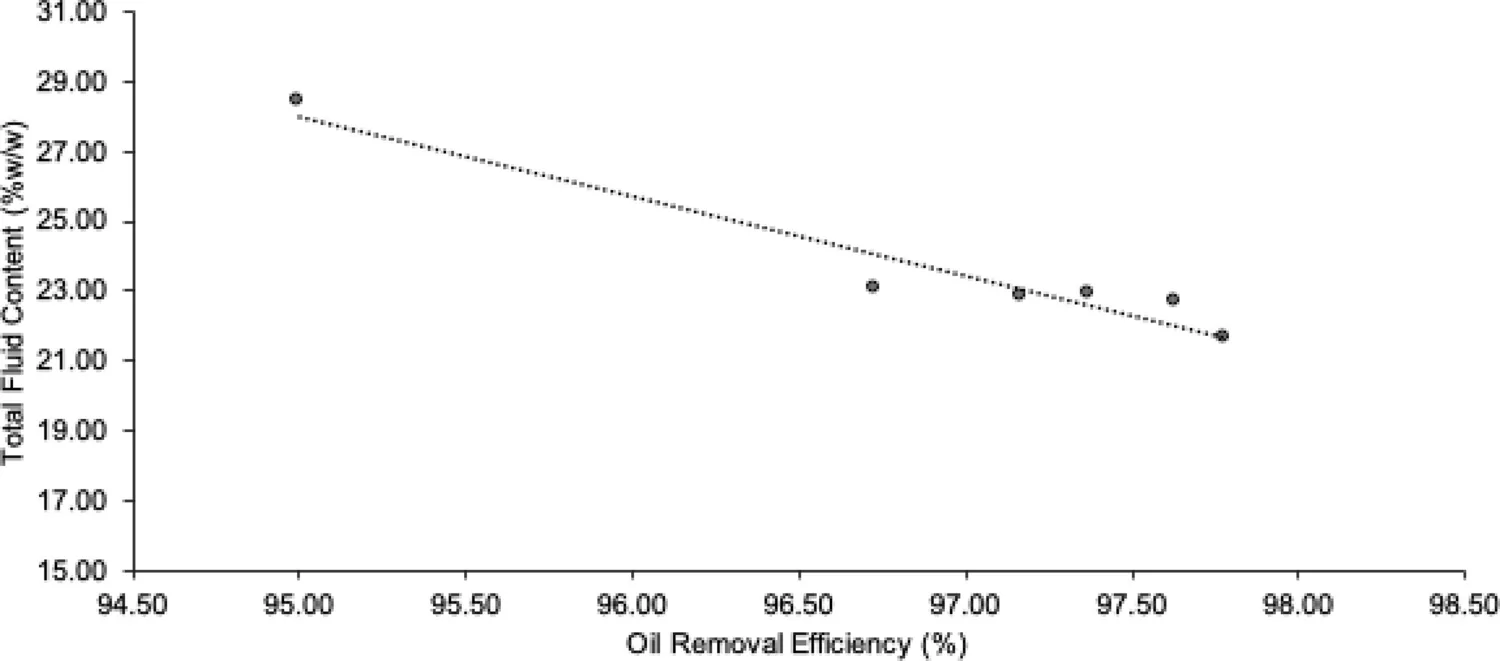
Relationship between initial total fluid content (oil + water) and oil removal efficiency for water-based drill cuttings treated using the ATP prototype

The effect of total fluid content (combined oil and water) on removal efficiency is shown in Fig. 7. A slight decrease in efficiency is observed as fluid content increases. Samples with total fluid content above ~ 23% w/w exhibited marginally lower oil removal efficiency compared to those with lower fluid content.

This trend can be attributed to thermodynamic and transport limitations during thermal desorption. At higher fluid contents, rapid heating may form insulating vapour layers around oil droplets, reducing effective heat transfer. This phenomenon, analogous to the Leidenfrost effect, has been reported in similar thermal systems (Noor et al. 2018).

Although based on a limited dataset, this trend is consistent with thermodynamic expectations and suggests that controlling total fluid content, including through pre-drying or moisture control, could enhance process performance. These findings support the optimisation of feed conditions and operating parameters to maximise oil removal efficiency in continuous treatment systems.

### Thermogravimetric analysis (TGA)

**Fig. 8:**
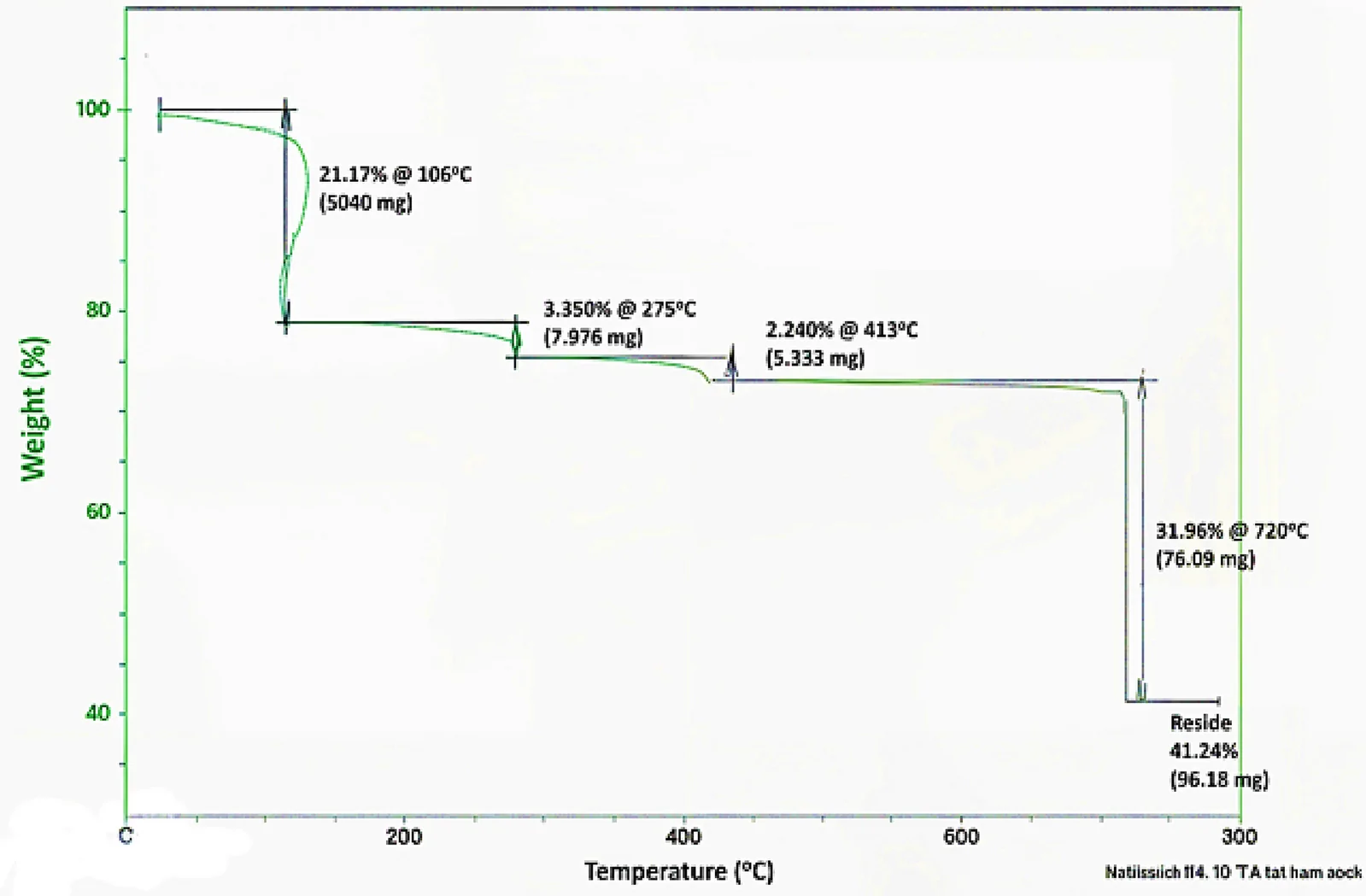
TGA curve of ATP-treated drill cuttings (Sample 1)

Thermogravimetric analysis (TGA) was conducted to characterise the thermal behaviour of the treated drill cuttings and assess the residual volatile fraction after treatment. For Sample 1 (Fig. 8), a major mass loss of approximately **21.17%** was observed at ~ **106 °C**, corresponding to the removal of water and light volatile hydrocarbons.

This result indicates that most volatile contaminants are removed at relatively low temperatures, confirming the efficiency of the hybrid microwave-infrared process. Only minor additional mass losses were observed at higher temperatures (275–413 °C), suggesting that the remaining material consists primarily of thermally stable inorganic components.

The absence of significant volatile loss below 400 °C after treatment is consistent with the low residual hydrocarbon content determined by the independent oil, TPH and TOG analyses. These findings also suggest that the process operates at lower thermal demand than conventional high-temperature thermal desorption methods, although further comparative studies are required.

This thermal profile is not only a validation of treatment completeness but also a demonstration of safe, low-discharge temperatures and environmentally compliant residue behaviour. In ATP, microwave and infrared heating penetrate the matrix volumetrically, rapidly driving off moisture and releasing embedded oil droplets without overheating or structural degradation.

### Process efficiency and practical implications

The hybrid microwave-infrared system demonstrated stable operation with efficient energy utilisation. Microwave heating enabled rapid volumetric energy transfer, while infrared heating improved surface heat distribution, resulting in uniform and effective treatment. The incorporation of a vapour recovery and gas recirculation system further enhanced process efficiency by recovering oil and reducing emissions. The ability to operate at relatively low temperatures while achieving high removal efficiencies highlights the potential of this technology for energy-efficient, continuous treatment of offshore drill cuttings. Overall, the results demonstrate that the hybrid ATP process provides a viable and scalable solution for drill cuttings management, combining high treatment efficiency with operational and environmental advantages.

## Conclusion

This study demonstrates that the hybrid microwave-infrared thermal desorption (ATP) process effectively treats oil-contaminated drill cuttings under inert conditions. The system consistently achieved oil removal efficiencies above 95% (up to 97.8%), reducing oil-on-cuttings to below the regulatory limit of 1 wt.%. Significant reductions in TPH and TOG (> 96%) further confirm the process’s effectiveness. Thermogravimetric analysis indicates that most volatile components are removed at relatively low temperatures (< 110 °C), suggesting a potential for lower thermal demand than conventional high-temperature treatments. The system also maintained stable performance across varying feed compositions, highlighting its robustness for handling heterogeneous offshore waste streams. Overall, the ATP system represents a promising and scalable solution for offshore drill cuttings management, combining high treatment efficiency with improved environmental performance. Future work should focus on full-scale validation and detailed energy and economic assessments.

## Data Availability

The datasets analysed during the current study are available from the corresponding author on reasonable request.
